# Peptide-based PET imaging of the tumor restricted IL13RA2 biomarker

**DOI:** 10.18632/oncotarget.16549

**Published:** 2017-03-24

**Authors:** Kiran Kumar Solingapuram Sai, Anirudh Sattiraju, Frankis G. Almaguel, Ang Xuan, Stephanie Rideout, Rahul S. Krishnaswamy, JoAnn Zhang, Denise M. Herpai, Waldemar Debinski, Akiva Mintz

**Affiliations:** ^1^ Department of Radiology, Wake Forest School of Medicine, Winston-Salem, NC, USA; ^2^ MicroPET/CT Imaging Section, TriFoil Imaging, Chatsworth, CA, USA; ^3^ Department of Cancer Biology, Brain Tumor Center of Excellence, Wake Forest School of Medicine, Winston Salem, NC, USA

**Keywords:** positron emission tomography (PET), high-grade glioma, doxycycline, biodistribution, IL13RA2

## Abstract

Peptides that target cancer cell surface receptors are promising platforms to deliver diagnostic and therapeutic payloads specifically to cancer but not normal tissue. IL13RA2 is a tumor-restricted receptor found to be present in several aggressive malignancies, including in the vast majority of high-grade gliomas and malignant melanoma. This receptor has been successfully targeted for diagnostic and therapeutic purposes using modified IL-13 ligand and more recently using a specific peptide, Pep-1L. In the current work, we establish the *in vitro* and *in vivo* tumor binding properties of radiolabeled Pep-1L, designed for tumor imaging. We radiolabeled Pep-1L with Copper-64 and demonstrated specific cell uptake in the IL13RA2-over expressing G48 glioblastoma cell line having abundant IL13RA2 expression. [^64^Cu]Pep-1L binding was blocked by unlabeled ligand, demonstrating specificity. To demonstrate *in vivo* tumor uptake, we intravenously injected into tumor-bearing mice and demonstrated that [^64^Cu]Pep-1L specifically bound tumors at 24 hours, which was significantly blocked (3-fold) by pre-injecting unlabeled peptide. To further demonstrate specificity of Pep-1L towards IL13RA2 *in vivo*, we exploited an IL13RA2-inducible melanoma tumor model that does not express receptor at baseline but expresses abundant receptor after treatment with doxycycline. We injected [^64^Cu]Pep-1L into mice bearing IL13RA2-inducible melanoma tumors and performed *in vivo* PET/CT and post-necropsy biodistribution studies and found that tumors that were induced to express IL13RA2 receptor by doxycycline pretreatment bound radiolabeled Pep-1L 3-4 fold greater than uninduced tumors, demonstrating receptor specificity. This work demonstrates that [^64^Cu]Pep-1L selectively binds hIL13RA2-expressing tumors and validates Pep-1L as an effective platform to deliver diagnostics and therapeutics to IL13RA2-expressing cancers.

## INTRODUCTION

Peptides that target cancer cell surface receptors are a promising platform to deliver diagnostics and therapeutics specifically to transformed but not normal tissue [1, 2]. PET/CT imaging using radiolabeled peptides can serve not only as a diagnostic test in itself [3] but also to stratify patients who express the particular biomarkers targeted by molecular therapies [4]. Examples of this PET-based stratification strategy were demonstrated to *a priori* predict response to common biomarker targeted therapies, including the estrogen receptor in breast cancer [4, 5]. Furthermore, PET-based imaging can expedite clinical development of targeted therapeutic strategies by demonstrating *in vivo* ligand targeting, biodistribution and kinetics [6].

The goal of this work was to utilize PET/CT to demonstrate the feasibility of using a peptide-based targeted approach to image interleukin-13 receptor α2 (IL13RA2) expression *in vivo* for the purpose of translating this promising platform to deliver diagnostic and therapeutic payloads. We previously identified IL13RA2 as an attractive molecular target that is highly overexpressed in glioblastoma (GBM) but not in normal brain [7–14]. GBM is the most common primary brain cancer and is defined by high morbidity and almost inevitable mortality [15, 16]. The median survival is less than 17 month with 5-y survival rate of <10%. Many research studies are currently being carried out to improve the diagnostic and therapeutic strategies for GBM [16–18]. One such strategy is to develop GBM specific biomolecular agents that target IL13RA2 and are used as potential diagnostic markers, imaging tracers and drug candidates [19–21]. In addition to brain cancer, IL13RA2 has also been reported to be highly expressed in other deadly malignancies, including melanoma, head and neck, ovarian and pancreatic cancers [22–24]. We and others have designed and developed a number of molecular targeted therapies based on IL13 and its derivatives that have shown their efficacy in preclinical models of various cancers [13, 19, 20, 25–29]. In fact, our targeted IL13-based agents were recently used as targeting ligand in chimeric antigen receptor (CAR)-engineered T cells that are in clinical trials [30]. Early results from this trial have reported safe and even remarkable results, including a case of a GBM patient experiencing regression of all intracranial and spinal tumors [30]. As this and other types of therapies are implemented against IL13RA2 in peripheral cancers that widely express IL13RA2, it is critical to develop a molecular imaging technique to confirm uniform biomarker expression for patient stratification. Furthermore, such a ligand can be used as a platform to deliver therapeutic radiation and targeted chemotherapy. Peptide-based approaches offer an advantage due to their small size and fast clearance, which has been shown to potentially increase the tumor-to-background ratio [31]. Furthermore, the ease of producing cGMP grade peptides is significantly easier than manufacturing properly folded targeted IL13 derivatives. Therefore, the focus of this study is to radiolabel Pep-1L, a novel peptide isolated from a hepta-peptide library that specifically binds to IL13RA2 [7, 32, 33] and determine its *in vitro* and *in vivo* tumor binding properties [34]. For peptide-based delivery platforms, Copper 64 (Cu-64) has become an attractive radionuclide in the development of a wide range of radiopharmaceuticals for PET due to its β+ emission, high specific activity, and half-life of 12 hours, which matches the kinetics of peptides [35]. Cu-64 easily binds to peptides through standard chelators [35, 36]. Therefore, to validate Pep-1L as an *in vivo* IL13RA2 targeting platform, we utilized PET/CT molecular imaging to visualize real-time Cu-64 labeled Pep-1L binding to a standard IL13RA2-expressing tumor. Furthermore, we produced a novel inducible human (h) IL13RA2-expressing melanoma tumor model to demonstrate specificity of Pep-1L to IL13RA2.

## RESULTS

### Radiolabeling Pep-1L with Cu-64

**Figure 1:**
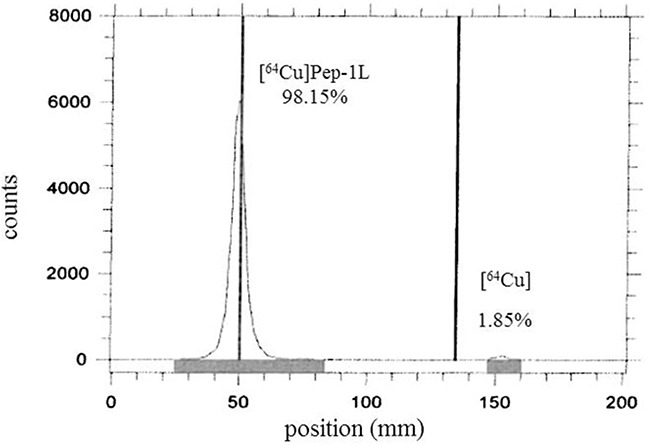
RadioTLC analysis of [^64^Cu]Pep-1L Analysis demonstrates 98.15% radiochemical purity (5 mM EDTA solution as mobile phase on an iTLC plate).

Extensive past studies involving radiolabeled peptides have demonstrated that 1,4,7-triazacyclononane-1,4,7-triacetic acid (NOTA) is an optimal way to radiolabel peptides. It forms a 6-coordinate prismatic complex with Cu(II) by coordinating the lone pairs of the three nitrogen atoms and the three carboxylate groups of the chelator [35–38]. Therefore, we utilized NOTA conjugated Pep-1L to radiolabel with Cu-64 using standard methodologies. NOTA conjugated Pep-1L was radiolabeled with Cu-64 in 0.1M NH_4_OAc buffer (pH 5.5) with > 98% radiochemical purity at 75°C for 1 h (n=15) (Figure 1). The R_f_ of the final radiolabeled product was ~0.13 while the unreacted Cu-64 was ~0.95 on iTLC strips with 5mM EDTA solution as mobile phase. The radiolabeling of Pep-1L with Cu-64 was tested over a wide range of aqueous buffer solutions with pH ranging from 4.0 to 7.5 and the highest radiochemical purity was achieved at pH 5.5. The radiolabeled peptide [^64^Cu]Pep-1L was used without any additional purification for both *in vitro* and *in vivo* studies.

### Serum stability of [^64^Cu]Pep-1L

**Figure 2:**
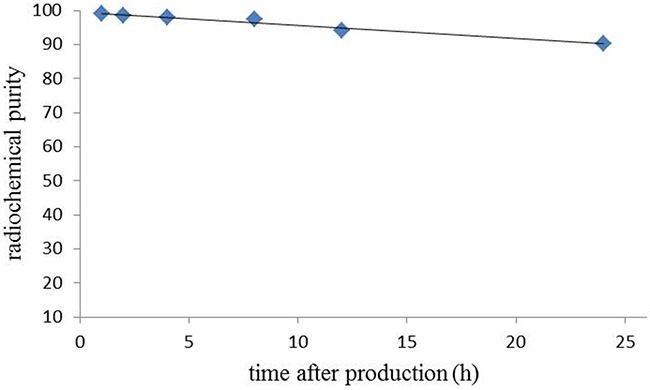
*In vitro* serum stability of [^64^Cu]Pep-1L Reverse-phase C18 radio-TLC analysis of [^64^Cu]Pep-1L over a 24 hour period post production.

The *in vitro* serum stability of the radiotracer [^64^Cu]Pep-1L in human serum was performed at different time points and was analyzed through radioTLC spotting (Figure 2). At 24 h post synthesis of [^64^Cu]Pep-1L, ~90% of the radioactive tracer remained intact. This data indicates that [^64^Cu]Pep-1L has good serum stability and suitable for *in vivo* use.

### *In vitro* cell uptake of [^64^Cu]Pep-1L in IL13RA2-expressing tumor cells

**Figure 3:**
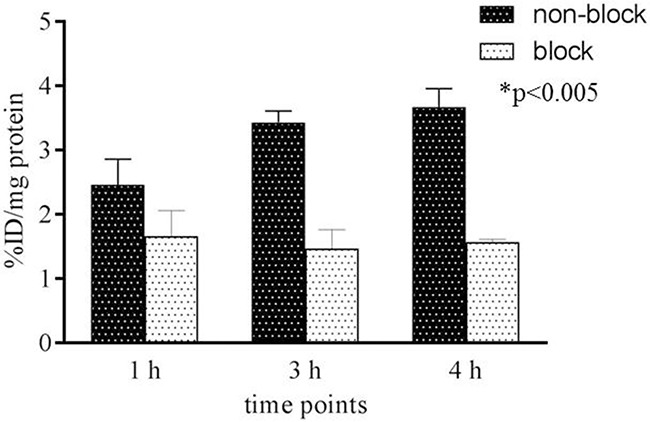
Cell uptake of [^64^Cu]Pep-1L Uptake of [^64^Cu]Pep-1L in IL13RA2-expressing G48 cells after 1, 3, and 4 h of exposure. Receptor blockade experiments were performed to demonstrate specificity by exposing cells to 50x excess unlabeled peptide, 15 min prior to adding radiolabeled peptide to saturate binding sites. The data were expressed as % injected dose (ID)/mg of protein present in each well with *p* values ≤ .005 considered statistically significant (n=6).

An *in vitro* cell uptake assay was performed on G48 human glioblastoma cells with and without unlabeled Pep-1L as blocking agent to demonstrate the specificity and binding efficiency of [^64^Cu]Pep-1L. Previously, we reported that this cell line contains about 4,000,000 IL13RA2 binding sites [13, 20, 26, 33]. [^64^Cu]Pep-1L bound these IL13RA2-expressing cells, which was significantly inhibited by binding site blockade with unlabeled Pep-1L (Figure 3). These *in vitro* uptake data demonstrate binding and specificity of [^64^Cu]Pep-1L towards IL-13RA2-expressing cancer cells.

### Biodistribution studies in mice bearing human IL13RA2-expressing xenografts

**Figure 4:**
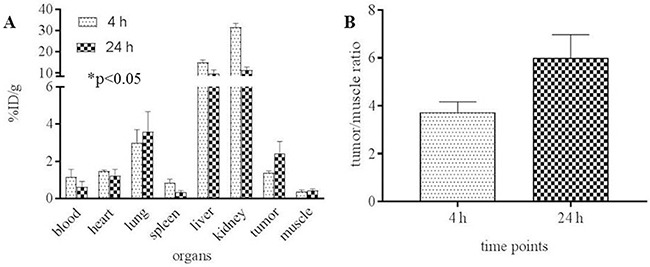
*In vivo* properties of [^64^Cu]Pep-1L **(A)** biodistribution and **(B)** tumor-to-muscle (target: nontarget) ratio of [^64^Cu]Pep-1L in mice bearing IL13RA2-expressing tumor xenografts 4h and 24h post injection (n=4) with *p* values ≤ .05 considered statistically significant.

Biodistribution studies with [^64^Cu]Pep-1L were performed in mice bearing IL13RA2-expressing G48 tumors (n=4) to confirm *in vivo* binding. [^64^Cu]Pep-1L displayed rapid clearance from most of the major organs from 4 h to 24 h post injection. However, tumor uptake steadily increased from 4 h to 24 h (Figure 4A) with %ID/g of 1.38±0.09 (4 h) and 2.43 ± 0.63 (24 h). The uptake ratios for target to non-target (tumor: muscle) increased from 3.7 to 6.0 from 4 h to 24 h (Figure 4B). Importantly, the tumor uptake was significantly blocked (5-fold) in mice pretreated with unlabeled peptide (n=4) after 24 h post injection i.e., %ID/g = 2.43±0.626 in the nonblockade group Vs. 0.49 ± 0.128 in the blockade group, demonstrating radiotracer specificity (Table 1).

**Table 1:** Biodistribution of [^64^Cu]Pep-1L in tumor bearing mice: non-blockade and blockade 24 h post injection with *p* values ≤ .05 considered statistically significant (n=4)

Organs	24 h non-blockade (%ID/g ± SD)	24 h blockade (%ID/g ± SD)
Heart	1.21 ± 1.012	1.86 ± 1.012
Blood	0.62 ± 0.579	1.04 ± 0.793
Lung	3.58 ± 1.078	3.41 ± 0.781
Liver	9.86 ± 1.593	6.59 ± 0.395
Kidney	17.45 ± 1.412	9.75 ± 2.140
Tumor	2.43 ± 0.626	0.49 ± 0.128
Muscle	0.41 ± 0.038	0.345 ± 0.164

### [^64^Cu]Pep-1L specifically binds doxycycline induced B16F10-Tet-hIL13RA2 tumors *in vivo*

**Figure 5:**
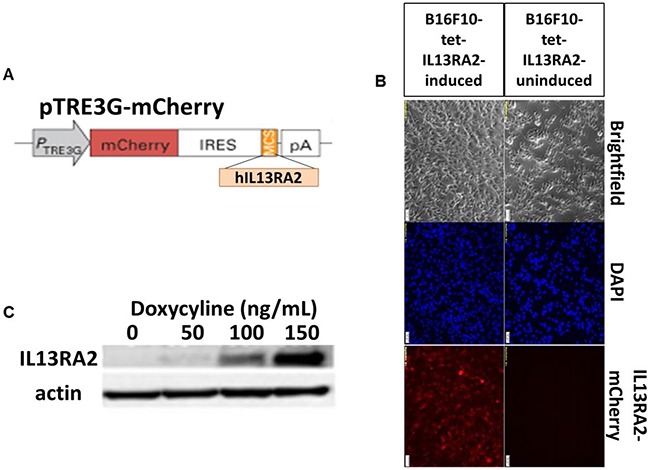
Characterization of inducible hIL13RA2 expressing cells **(A)** Scheme of inducible hIL13RA2 expressing plasmid. The receptor sequence was cloned into the pTRE3G vector that contains a tetracycline inducible promoter (TRE3G), which drives expression of the mCherry fluorescent reporter and hIL13RA2 **(B)** Induced mCherry fluorescence tag visualized under a fluorescent microscope. mCherry fluorescence is seen only in doxycycline induced clone 18 B16F10 cells *in vitro*. Non-induced clone 18 B16F10 cells show lack of mCherry fluorescence. Doxycycline activated production of hIL13RA2 protein in B16F10 clone 18 cell line. **(C)** Western blotting using antibody for hIL13RA2 shows proportionally increasing levels of hIL13RA2 as concentration of doxycycline increases.

To further confirm the *in vivo* specificity of [^64^Cu]Pep-1L to IL13RA2, we created a novel IL13RA2-inducible cell line. B16F10 murine melanoma cells, which do not endogenously express IL13RA2, were transfected with a tetracycline inducible plasmid expressing both mCherry and hIL13RA2 (Figure 5A). Upon induction with doxycycline, mCherry fluorescence (610 nm) was observed *in vitro*, confirming successful transfection and induction of reporter gene (Figure 5B). Single colonies were generated by serially diluting cell suspensions of B16F10-Tet-hIL13RA2 cells and a high IL13RA2-expressing doxycycline inducible clone was selected for further studies. Importantly, we observed a proportional increase hIL13RA2 expression with increasing concentrations of doxycycline (Figure 5C).

**Figure 6:**
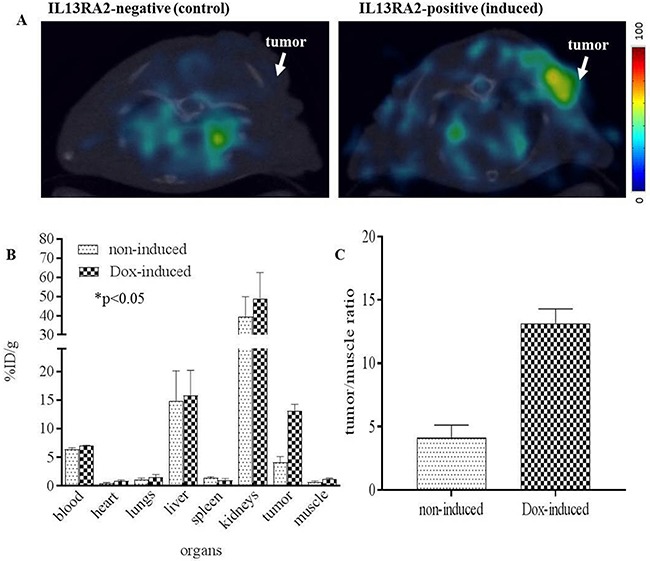
PET/CT of [^64^Cu]Pep-1L targeting IL13RA2 expressing tumors Representative axial fused microPET/CT images obtained after injection of [^64^Cu]Pep-1L in a novel doxycycline inducible IL3RA2 tumor model. **(A)** PET/CT of IL13RA2-induced and uninduced tumor-bearing mice at 4 h post injection. **(B)** Post-PET biodistribution analysis shows [^64^Cu]Pep-1L specifically accumulates in hIL13RA2 expressing induced B16F10 tumors with absolute values in %ID/g with with *p* values ≤ .05 considered statistically significant (n=4) and **(C)** tumor/muscle ratios of [^64^Cu]Pep-1L accumulation in hIL13RA2-expressing B16F10 tumors compared to uninduced control tumors.

To demonstrate *in vivo* receptor specificity of [^64^Cu]Pep-1L, hIL13RA2-induced B16 tumors were generated by injecting mice flanks with B16F10-Tet-hIL13RA2 cells. 7 days after tumor cell implantations, mice were divided into induced and uninduced (control) groups (n=4/group). To induce hIL13RA2 expression, doxycycline was administered intraperitoneally (2.5 mg/mouse) every 12 hours for 2 days [39, 40]. After IL13RA2-induction, [^64^Cu]Pep-1L was intravenously injected to mice bearing induced or control (uninduced) subcutaneous tumors and microPET/CT imaging was performed 4 h post intravenous injection. ROI analysis of micro PET/CT imaging data showed that [^64^Cu]Pep-1L accumulation was ~3-fold greater in doxycycline-induced B16F10-Tet-hIL13RA2 tumor bearing mice compared to non-induced ones (Figure 6A) demonstrating specificity of [^64^Cu]Pep-1L to IL13RA2 expressing tumors. These results were corroborated in the post-PET biodistribution studies that demonstrated significant tumor uptake in the hIL13RA2-induced tumors compared to the non-induced controls (Figure 6B and 6C). [^64^Cu]Pep-1L demonstrated ~4.0 fold greater accumulation in the tumor of doxycycline-induced tumor bearing mice (%ID/g = 14.09 ± 0.246) compared to non-induced tumor bearing mice (%ID/g = 4.179 ± 0.007) (Figure 6B). Other notable findings were that there was radiotracer accumulation in liver and kidney in both induced and control animals, which are primary organs for peptide clearance. These biodistribution results were consistent with the microPET imaging data and confirmed *in vivo* targeting of Pep-1L.

## DISCUSSION

The ability to target a specific receptor that is highly expressed on the surface of cancer cells creates the opportunity to use targeted peptides as highly selective platforms to deliver diagnostic and therapeutic payloads to cancer [31]. Therefore, in this work we used translational PET imaging to demonstrate the *in vivo* targeting of Pep-1L, a peptide reported to target IL13RA2 [7], an attractive tumor-associated biomarker that we and others have demonstrated to be present on a number of deadly malignancies, including GBM and melanoma [13, 41, 42].

Pep-1L was conjugated with NOTA, a chelator that can form a six-coordinate complex with Cu-64 that results in a high stability constant (log K=21.6) by CPC scientific Inc. [34]. This is more stable that than other commonly used chelators, such as TETA or DOTA [43]. The Cu-64 radiolabeling conditions for Pep-1L were well optimized in this work for high radiochemical purity of >98%, without any need for additional purification methodologies with a specific activity of 32-35 MBq/μg (Figure 1). Importantly, [^64^Cu]Pep-1L demonstrated *in vitro* serum stability (~90% intact for 24 h), demonstrating translational significance (Figure 2).

To demonstrate *in vitro* cell uptake, we performed binding studies on the G48 GBM cell line that we previously established to express 4,000,000 IL13RA2 binding sites. These studies demonstrated high tumor cell uptake, which was significantly blocked by unlabeled Pep-1L, showing specificity (Figure 3). Importantly, uptake was maintained over time and was still present in high levels at 4 h, which is expected due to known internalization properties of Pep-1L, indicating the suitability of this peptide as a carrier of potential therapeutics. We initially confirmed [^64^Cu]Pep-1L's tumor binding properties *in vivo* in mice bearing IL13RA2-expressing G48 tumors (Figure 4) and found specific binding at 24 hours, which was blocked (3-fold blockade) by pre-injecting unlabeled peptide (Table 1). There was minimal (%ID/g ~0.013 ± 0.005 at 4 h and ~ 0.009 ± 0.003 at 24 h) or no radiotracer uptake in the brain at these delayed times, indicating no peptide residence or binding. However, further studies are currently being performed to evaluate dynamic uptake in normal brain as well as in brains containing orthotopic tumors. To further validate the specificity of [^64^Cu]Pep-1L *in vivo*, we produced a novel inducible hIL13RA2 expressing murine melanoma cell line that we demonstrated expresses IL13RA2 only after induction with doxycycline (Figure 5). Importantly, doxycycline-induced B16F10-Tet-IL13RA2 subcutaneous tumors demonstrated significantly more [^64^Cu]Pep-1L uptakeon PET/CT imaging compared to uninduced controls (Figure 6). These results were confirmed on Post-PET biodistribution analysis (Figure 6) and further validate the specific binding of [^64^Cu]Pep-1L to IL13RA2.

Receptor targeted platforms like Pep-1L are increasingly being used to target chemotherapies and radiation to cancer [44, 45]. IL13RA2 is especially attractive due to its ubiquitous expression on GBM, melanoma and other cancers. Furthermore, IL13RA2 has been shown to be internalized together with its ligand and attached payload [7, 46]. This property of Pep-1L was initially described by Pandya et al [33] but confirmed in our current study, as we saw a continued increase in [^64^Cu]Pep-1L uptake from 1 to 4 hours. In addition to demonstrating tumor targeting and therapeutic potential of Pep-1L, we also developed a translational companion diagnostic that can be used to demonstrate real-time IL13RA2 expression in tumors. Knowing each tumor's IL13RA2 expression profile can help personalize IL13RA2-targeted therapies to patients that have tumors that express significant amounts of IL13RA2.

## CONCLUSION

This work demonstrates that [^64^Cu]Pep-1L selectively binds hIL13RA2-expressing tumors and validates Pep-1L as an effective platform to deliver diagnostics and therapeutics to IL13RA2-expressing cancers.

## MATERIALS AND METHODS

### Chelators and chemicals

The sequence of the Pep-1L peptide was as follows: H-Ala-Cys-GlyGlu-Met-Gly-Trp-Val-Arg-Cys-Gly-Gly-Gly-Ser-LCLys-Lys(biotin)-NH_2_. Pep-1L conjugated to NOTA was purchased from CPC scientific ltd, Sunnyvale, CA. All reagents were purchased from Sigma-Aldrich, St. Louis, MO and were used without additional purification. Reversed phase TLC plates were purchased from Whatman Inc, Florham Park, NJ.

### Radiolabeling procedure

Copper-64 (^64^Cu: t_1/2_=12.7 h) was purchased from Washington University in St. Louis. The custom Peptide-NOTA was conjugated by CPC scientific Inc. Custom Peptide-NOTA was radiolabeled with ^64^Cu according to the previously reported methods [35, 36, 38, 47]. Briefly, 20 μg of NOTA-Peptide was dissolved in 0.1 M ammoniumacetate (NH_4_OAc) aqueous buffer solution pH 5.5. ^64^CuCl_2_ was converted to ^64^Cu(OAc)_2_ (^64^Cu-acetate) by dissolving in 100 μL of 0.1 M NH_4_OAc pH 5.5. The resultant ^64^Cu(OAc)_2_ was then added to the conjugated NOTA-Peptide aliquot in an Eppendorf tube and heated at 75°C for 1h. Radiochemical purity was determined by radio-TLC (MK-C18 reversed phased TLC plates). Around ~1 μL of the reaction mixture was applied on the C18-reversed phase TLC plate and developed with 10% NH_4_OAc: MeOH (30:70) as the mobile phase. After the completion of reaction (through TLC analysis), the reaction mixture was quenched with 5mM EDTA aqueous solution and stirred for an additional 15 min at 30°C. The final product was passed through 0.22 μm filter and re-analyzed by iTLC strips with 5mM EDTA solution as mobile phase. The TLC plates were scanned on a BioScan Imaging Scanner.

### Serum stability

The *in vitro* serum stability of [^64^Cu]Pep-1L was performed using human serum (Sigma Aldrich) following previously published methods [38, 48]. Approximately 0.037 MBq/10 μL of [^64^Cu]Pep-1L was added to the serum vial, to a final volume of 100 μL and incubated at 37°C [35]. 1 μL of the sample mixture was removed and spotted on a reverse C18-TLC plate at 1 h, 2 h, 4 h, 18 h and 24 h after the radiotracer synthesis and the percentage of bound Cu-64 was determined through radioTLC analysis as mentioned above [48].

### Cell culture

Human glioblastoma cell line G48 and murine melanoma cell line (ATCC) B16F10 were cultured in DMEM (Gibco) base media supplemented with 10% (v/v) Fetal Bovine Serum (Invitrogen) and 1% Anti-anti (Gibco). Following washing, cells were spun down, resuspended in growth media and counted using a hemocytometer.

### *In vitro* cell uptake assay

The *in vitro* reactivity, binding affinity and specificity for [^64^Cu]Pep-1L were determined using human GBM cell line G48, following previously reported methods [34, 38, 49]. 1 × 10^5^ cells were then seeded into each well of a 6-well culture. G48 cells were incubated overnight at 37°C with 5% CO_2_ in an incubator. On the day of the assay, fresh solution of peptide was made at a concentration of 5 μM in the respective cell media and was used as the blocker solution. The blocker solution was added 15 min prior to addition of radiotracer. G48 cells were incubated with [^64^Cu]Pep-1L (0.0185 MBq/well) for 1h, 3h and 4h (n=3) at 37°C. The cell uptake assays were initiated by rinsing the cells with 2 × 2 mL of the phosphate buffer at room temperature. Uptake was allowed to proceed for selected time periods and then terminated by rinsing the cell wells with 1 mL of the ice-cold buffer solution. Residual fluid was removed by pipette, and 200 μL of 0.1% aqueous sodium dodecylsulfate lysis buffer solution was added to each well. The plate was then agitated at room temperature and 1 mL of the lysate was taken from each well for gamma counting [49]. The radioactivity was counted using the Wallac 1480 Wizard gamma counter (Perkin Elmer, Turku, Finland). Additional 20 μL aliquots were taken in triplicate from each well for protein concentration determination using the Pierce bicinchoninic acid protein assay kit method (Rockford, IL).

The uptake data in each sample from each well and the standard counts for each condition were expressed as counts per minute (cpm) of activity and were decay corrected for elapsed time. The cpm values of each well were normalized to the amount of radioactivity added to each well and the protein concentration in the well and expressed as percent uptake relative to the control condition. The data were expressed as %ID/mg of protein present in each well with *p* values ≤ 0.005 considered statistically significant.

### Biodistribution studies in tumor bearing mice

Athymic nude mice (Taconic Farms) were housed in a pathogen-free facility of the Animal Research Program at Wake Forest School of Medicine under a 12:12-h light/dark cycle and fed ad libitum. All animal experiments were conducted under IACUC approved protocols in compliance with the guidelines for the care and use of research animals established by Wake Forest Medical School Animal Studies Committee. IL13RA2-expressing G48 tumors cells (1 × 10^5^ cells suspended in 10 μL) were implanted in the left flank of nude mice (25-30 g) as described previously [10, 13]. The presence of viable tumors was confirmed through bioluminescence imaging. Standard biodistribution studies were performed in the same nude mice with flank tumors (n=4) at 10-15 days after tumor implantation. Mice were anesthetized with 1% isoflurane-oxygen and approximately 3.7- 4.6 MBq of [^64^Cu]Pep-1L was administered via tail vein injection. To demonstrate specific binding, blocking experiments were performed on the mice from the 24 h time point group (n=4/group). Peptide concentration of 15 mg/kg was used as the blocking agent and was intravenously injected via tail vein 15 min prior to the radiotracer. The tumor and organs of interest were dissected and gamma counted using Wallac 1480 Wizard gamma counter (Perkin Elmer, Turku, Finland). The concentration of the radioactivity in the tumor and organs were expressed in percentage injected dose per gram (%ID/g) of radioactivity with *p* values ≤ .05 considered statistically significant and uptake ratio of tumor-to-muscle was calculated.

### Generation and analysis of IL13RA2-inducible B16F10 melanoma tumors

To create the inducible system, B16F10 melanoma cells were stably transfected with pCMV-Tet3G (Clontech), which encodes for the advanced Tet-On 3G transactivator element under constitutive CMV expression using standard cloning methods [50]. Cells were then stably transfected with the pTRE3G-mCherry-IRES-IL13RA2 plasmid, which encodes proportional expression of mCherry (bottom) and human IL13RA2 under the Tet-On promoter. Single colonies were isolated using serial dilution in 96 well plates and clones were analyzed for IL13RA2 expression via Western blot using methods we previously described [50]. For analysis of RFP expression, an inverted motorized fluorescent microscope (Olympus IX81) with an Orca-R2 Hamamatsu CCD camera (Hamamatsu) and a laser scanning confocal microscope (Olympus FluoView1200) were used for image acquisition. Camera drive and acquisition were performed using a MetaMorph Imaging System (Olympus) and FluoView 4.2 software were used for image acquisition.

### Subcutaneous implantation of B16F10 cell line

B16F10 murine melanoma cell line was cultured in DMEM base media. Cells were harvested using trypsin-EDTA (Gibco) after removing DMEM media from flasks and washing residual with DPBS (Lonza). Cell number was calculated using a hemocytometer and required number of B16F10 cells was suspended in DMEM media. The cell suspension was then mixed with growth factor reduced matrigel (Corning) in a 1:1 ratio and placed on ice. Athymic nude mice were anesthetized using isoflurane and injected with 1 million cells (in matrigel) subcutaneously near the left shoulder using 28G insulin syringes while being placed on a heating pad. Mice were then earmarked for identification and placed back in their cages.

### Doxycycline administration for IL13RA2-induction in mice bearing subcutaneous B16F10-Tet-hIL13RA2 tumors

Tumor bearing athymic nude mice were grouped into two groups (n=4). Two days prior the radiotracer injection and PET study, doxycycline (2.5 mg/mouse) was administered intraperitoneally to one group of mice (n=4) every 12 h.

### PET/CT and post-necropsy biodistribution studies in mice bearing subcutaneous B16F10-Tet-IL13RA2 tumors

Mice bearing subcutaneous tumors were grouped into two groups (n=4) and placed in an induction chamber containing ~2% isoflurane/oxygen then secured to a custom double bed for placement of tail vein catheters. Anesthesia was maintained via nose-cone at ~1% isoflurane/oxygen for the dynamic imaging procedure. The mice were injected with 4.62-5.5 MBq [^64^Cu]Pep-1L and scanned for 20 min using TriFoil PET/CT scanner 4 h post injection. The regions of interest (ROIs) were generated for both dox-treated and non-induced mice from manually drawn regions of interests. After the completion of microPET data acquisition, mice were euthanized for a confirmatory biodistribution studies. Samples of tumor, blood, lung, liver, spleen, kidney, muscle and heart were harvested, weighed and then counted on the gamma counter with a standard dilution of the injectate. The percentage of the injected dose per gram of tissue (%ID/g) was calculated as described above with *p* values ≤ .05 considered statistically significant (n=4).
